# Simulated endocardial vegetation model highlights the complexity of high-inoculum infections among β-lactamase-producing organisms

**DOI:** 10.1128/aac.01340-25

**Published:** 2025-12-10

**Authors:** Andrew J. Fratoni

**Affiliations:** 1 Center for Anti-Infective Research and Development, Hartford Hospital 23893, Hartford, Connecticut, USA; University of Pennsylvania Perelman School of Medicine, Philadelphia, Pennsylvania, USA

**Keywords:** infective endocarditis, *Enterobacter*, beta-lactamases, beta-lactams

## Abstract

Conventionally, susceptibility testing and pharmacokinetic/pharmacodynamic relationships are determined using standard inoculum (i.e., 10^5^–10^6^ CFU). These may be poorly predictive of efficacy for high-inoculum infections, especially amongst β-lactamase-producing organisms. A. J. Kunz-Coyne, R. Gray, E. May, H. Curry, et al. (Antimicrob Agents Chemother 69:e01170-25, 2025, https://doi.org/10.1128/aac.01170-25) used a 96-h simulated endocardial vegetation model to describe pharmacodynamic efficacy, resistance emergence, and β-lactamase expression that resulted after clinically relevant exposures of antibiotics against three *Enterobacter cloacae* complex isolates, demonstrating that MIC values were often poorly predictive of efficacy in the model.

## COMMENTARY

*Enterobacter cloacae* complex (ECC) is not often high on the list of frequently encountered pathogens responsible for infective endocarditis. Unfortunately, reported treatment outcomes leave something to be desired (1). ECC is a key member of AmpC-producing gram-negative organisms (2). While MIC testing, *in vitro* time kill experiments, and *in vivo* pharmacokinetic/pharmacodynamic (PK/PD) infection models traditionally operate with starting inoculums from 10^5^ to 10^6^ CFU/mL, it is vital to consider how antibiotic exposure/response relationships might differ at higher bacterial burdens, such as those observed in deep-seated infections like endocarditis.

Kunz-Coyne and colleagues employed a 96-h simulated endocardial vegetation endocarditis model to describe pharmacodynamic efficacy, resistance emergence, and β-lactamase expression that resulted after clinically relevant, human-simulated exposures of cefepime, ertapenem, meropenem (with and without vaborbactam), and ceftazidime-avibactam were administered against three ECC isolates (3). All three isolates originated from the CDC and FDA Antimicrobial Resistance Isolate Bank. CDC 0008 and 0060 express ACT-15 and -89, respectively, AmpC β-lactamases that are inherent to ECC. CDC 0032 also expresses ACT-16 but additionally has other acquired β-lactamase genes, KPC-3 and TEM-1. Generally speaking, based on these genotypic profiles, cefepime or a carbapenem would be considered reasonable therapeutic options for 0008 and 0060, while one of the aforementioned BL/BLIs could be considered for 0032. However, considering the impact of a high-inoculum infection raises uncertainty and leads one to question whether standard antibiotic selections are best suited for these types of clinical scenarios.

In their endocarditis model, cefepime 2 g q8h as either 30-min intermittent infusion (II) or 3-h extended infusion (EI) failed to achieve bactericidal activity (i.e., ≥3 log_10_CFU/g reduction) at 96 h and resulted in multi-fold MIC increases in all three ECC isolates, while changes in gene expression were mixed. As would be expected based on the presence of KPC-3, ECC 0032 showed net growth on meropenem monotherapy, with an approximate 15-fold uptick in ACT-16 induction. Meropenem yielded reductions of −2.9 and −4.6 log_10_CFU/g against EC 0060 with II and EI, respectively, suggesting additional efficacy with prolonged infusion. In the same isolate, ertapenem demonstrated modest (−1.9 log_10_CFU/g) reductions at 96 h with an associated MIC increase.

Regarding the BL/BLIs that were tested, both meropenem-vaborbactam and ceftazidime-avibactam were bactericidal against ECC 0008 (ACT-15) with minimal change to both MIC and ACT expressions. The addition of vaborbactam to meropenem greatly improved killing (−3.6 log_10_CFU/g) against ECC 0032 (KPC), while ceftazidime-avibactam failed to achieve bactericidal activity (−1.2 log_10_CFU/g). Notably, there have been clinical reports of high-level treatment-emergent resistance to ceftazidime-avibactam due to modification of chromosomal AmpC in ECC and *Klebsiella aerogenes* (4, 5).

Studies like these are important to remind us that conventional *in vitro* susceptibility testing and PK/PD relationships determined using standard inoculum (i.e., 10^5^–10^6^ CFU) may fall short in translating to certain clinical scenarios. Breakpoints are determined based upon data primarily generated at these standard conditions, where “S” may provide a false sense of security when dealing with enzyme-mediated resistance and high-inoculum deep-seated infections that readily form biofilm. Indeed, aside from ertapenem and ECC 0008, none of the tested drug/bug combinations were categorically resistant using standard MIC methodology. Conducting adequately powered randomized controlled clinical trials (RCTs) for relatively rare and challenging conditions, such as ECC infective endocarditis, is an unreasonable expectation. In lieu of the gold-standard RCT-level of evidence, there remain some tools at our disposal. When Kunz-Coyne et al*.* conducted MIC testing using a higher inoculum of 10^7^ CFU/mL, it foreshadowed many of the failures seen in their 96 h endocarditis model. However, the inoculum effect of the modified MIC method did appear to overestimate the level of resistance relative to what was seen in the model. Even the BL/BLIs demonstrated a marked inoculum effect, yet achieved net reduction in the endocarditis model. This observation might be explained in part by the static nature of MIC testing and time-kill analyses. These methods rely on static drug concentrations, which do not reflect the physiologic reality in patients. Conversely, dynamic models, such as this endocarditis model, can simulate concentration-time profiles akin to what is observed clinically, with concentrations rising and falling accordingly with drug administration and subsequent clearance. This is likely critically important for the time-dependent killing of β-lactams and enzyme-mediated resistance.

Another consideration for these types of models is they often represent the efficacy of the antibiotic without the contribution of the immune system. Thus, this “worst-case scenario” may not be reflective of the drug’s efficacy in many clinical scenarios. An alternative model that may be able to better characterize the efficacy of antibiotics for infective endocarditis in the presence of a functioning immune system is the porcine infective endocarditis model, but it is often prohibitively expensive and resource intensive (6).

I applaud the efforts of Kunz-Coyne et al. (3) in highlighting the complexity of high-inoculum infections among β-lactamase-producing ECC. Similar types of experiments with alternative bacterial species producing both inducible and plasmid-mediated β-lactamases should be encouraged to gain more translational insights into optimizing the care of patients with challenging high-inoculum infections. As the authors point out, β-lactamase resistance does not occur in a vacuum and is frequently accompanied by porin loss and efflux-pump overexpression. These dynamics in high-inoculum infections should also be considered.

While optimizing antibiotic therapy is critically important for improving the outcomes of patients, let us not forget that the best “antibiotic” of them all is source control.
